# Correction: Acquisition and maintenance of disgust reactions in an OCD analogue sample: Efficiency of extinction strategies through a counter-conditioning procedure

**DOI:** 10.1371/journal.pone.0319260

**Published:** 2025-02-07

**Authors:** The PLOS One Editors

## Notice of Republication

This article [1] was republished on May 14, 2024, to address an issue identified post-publication. An updated version of S1 File is provided with this notice. Please download this article again to view the correct version.

## Supporting information

S1 File Original underlying data. (XLSX)
