# Supplementary material for: Acquisition and maintenance of disgust reactions in an OCD analogue sample: Efficiency of extinction strategies through a counter-conditioning procedure
Source: PLoS One. 2021 Jul 14;16(7):e0254592. doi: 10.1371/journal.pone.0254592 (PMC8279387; doi:10.1371/journal.pone.0254592)
Supplement: Attachment — Submitted filename: PONE-D-20_33124.rtf [file pone.0254592.s002.rtf]

This manuscript reports a novel replication and extension of Armstrong and Olatunji's (2017) study of disgust learning in contamination-based OCD. The article mostly replicated the findings in this paper, but with some interesting differences. The main limitation with this study is that it lacks an extinction procedure to which the counter-conditioning procedure could be compared. Thus, it is unclear to what extent the findings reflect the lack of the disgusting US following the CS during counter-conditioning, versus the presence of the pleasant counter-conditioning US. That said, the finding still makes some contribution by showing how the results of Armstrong & Olatunji (2017) replicate with a counter-conditioning procedure instead, and the authors note the need to add other treatment conditions in the future. Advice for improving the manuscript is provided below: 

Introduction:

“If” at beginning of abstract is confusing. 

This line is the confusion is a little awkward: “Desensitization to disgust handled by counter-conditioning…” Could the authors just use the term “counter-conditioning.”

I would avoid the term “Disgust desensitization.” For many, the term sensitivity refers to how much one is bothered by an emotional state, not how prone they are to experiencing it (the authors note this distinction).  
  Line 88: Typo?: “there is a compelling argument recent research has pointed out”

Line 97: “previously conceptualized disorders within the exclusive framework of anxiety” should be “disorders previously conceptualized within…”

Paragraph on line 107 too short

The paragraph beginning on line 111 needs revision. I found many of these proposed explanations for disgust resisting extinction to be hard to follow and not compelling. I would get right to the findings with taste aversion learning and the work of Garcia. Then transition to evaluative conditioning, which to my knowledge was first studied in taste preference (e.g., the “hedonic shift” of the flavor of sucrose after being paired with poison that causes nausea; see Bouton 2007 for summary). It might help to highlight the relation between distaste and disgust earlier in the introduction (disgust is an emotion that seems to be an elaboration of the distaste oral rejection reflex—same outputs mapped onto more abstract inputs). If the authors wish to argue that disgust is resistant to extinction because of EC, they need to do a better job explaining how EC is involved in disgust. Perhaps bring in Rozin et al. 1986, discuss the transfer of essences as a hedonic shift?

The paragraph on line 128 needs to be clarified. The section on EC right now is a little redundant in places. Also, some of the claims about EC and the CR becoming independent of the US-CS relation have been challenged. The description of EC in this paper seems a little outdated. 

 Methods:

I would suggest removing “dirt” from the group names—just the term contamination should suffice. 
 
Power

DPSS is propensity and sensitivity scale, not propulsion and sensibility.

The ratings do not seem retrospective, because they don't ask about feelings of disgust in the past, during the task. This is fine; I would just suggest not calling them “retrospective” ratings. 


Results:
Error in table 1: Genre % Female 

The term valence is confusing in this section. The measure is called the empirical valence scale, but it is used here to measure disgust, rather than the bipolar valence dimension of affect, so the outcome should be referred to as disgust not valence. 

The figures could use a y-axis label. It is clear that it is the ratings, but with the different min/max it is a little confusing. Also, I might try to produce better figures, as these figures have no error bars, have periods after the Y-axis values, and have counter-conditioning misspelled. 

Line 286: I did not follow this: “A t-test 287  reveals that only conditioned CS (CS +) was significantly assessed as causing disgust in the HDCC 288  group who associated the aversive properties of the CS from the acquisition phase t(43)=5.17, p=.02” 
Perhaps use holm-bonferroni procedure to increase power?

In the methods section, I did not complete understand this sentence: “Regression analyses was then conducted through a step by step procedure including the whole of the 7 sub-dimensional factors assessed by scales. “
Then, in the results section, I did not follow the related analyses. It would help to be more specific about the dependent variable in these analyses.


Discussion: 

The authors claims that “From a clinical point of view, exposure-based treatment incorporating counter-conditioning would thus be more effective than simple exposure treatment by addressing not only the predictive value  of the unconditioned stimulus occurrence but also by modifying the valence attributed to the conditioned stimulus.” However, as they note in the limitations, there is not direct comparison to an extinction procedure. In the limitations, I would be more explicit about needing to replicate the effect with an extinction comparison group, as other researchers have done.

One other limitation is statistical power. The group sizes are relatively small for detecting between-groups differences. This limitation applies to much of the conditioning literature.   
The authors note the need for other dependent variables in their conditioning research. They might consider eye tracking: e.g., Armstrong, T., Engel, M., Press, T., Sonstroem, A., & Reed, J. (2019). Fast-forwarding disgust conditioning: US pre-exposure facilitates the acquisition of oculomotor avoidance. Motivation and Emotion, 43(4), 681-695.


   
