# Supplementary material for: Acquisition and maintenance of disgust reactions in an OCD analogue sample: Efficiency of extinction strategies through a counter-conditioning procedure
Source: PLoS One. 2021 Jul 14;16(7):e0254592. doi: 10.1371/journal.pone.0254592 (PMC8279387; doi:10.1371/journal.pone.0254592)
Supplement: Attachment — Submitted filename: response to reviewer Final without TC.docx [file pone.0254592.s003.docx]

16 of April, 2021

Ref PONE-D-20-33124

Dear Editor,

We have carefully considered the Reviewers’ and Editor’s comments and have revised the manuscript accordingly. Below we provide a detailed explanation of how the manuscript was modified.

Reviewer #1:

*1) Though the authors used eight images representing landscapes (2), family (1), animals (2), baby (1), laughing children (2) for counter conditioning, they did not measure pleasantness valence to ask participants “How much do you find this picture pleasant?.” They should discuss possibility of difference in pleasantness valence between HDCC and LDCC and add the limitation of the study.*

**Response:** We agree with this comment.

We added in the limitation section

P.21 Line [ 8 ] « Then, we did not measure pleasantness valence of the stimuli used for our evaluative conditioning procedure. Indeed, OCD has been associated with increased levels of depression (Angelakis et al., 2015), with in turn has been associated with a reduced reactivity to emotional stimuli (Bysmla et al., 2008). Taken together the difference found in our study between HDCC and LDCC coud be explained by a reduced emotional reactivity to pleasantness between our two groups. »

**Angelakis I**, Gooding P, Tarrier N, Panagioti M.Suicidality in obsessive compulsive disorder (OCD): a systematic review and meta-analysis.Clin Psychol Rev. 2015 Jul;39:1-15. doi: 10.1016/j.cpr.2015.03.002.

Bylsma LM, Morris BH, Rottenberg J. A **meta**-**analysis** of emotional reactivity in major **depressive** **disorder**. Clin Psychol Rev. 2008 Apr;28(4):676-91. doi: 10.1016/j.cpr.2007.10.001

*2) The mean disgust valence scores during habituation in the report by Armstrong et al. (Behaviour Research and Therapy, Volume 93, June 2017, Pages 78-87) were 20.62 in HCC and 10.93 in LCC, respectively. In the current study, the mean disgust valence scores during habituation were 5.18 in HDCC and 1.26 in LDCC, respectively. The authors should discuss the big difference between their current study and the report by Armstrong et al. (2017) and add the limitation of the study.*

**Response:** We thank reviewer #1 for this comment. Indeed, the reason for the huge differences between our work and Armstrong’s one is probably (as you have noticed in the next comment) linked to the intensity of the disgusting US presented. However, and fortunately, the transfer of valence between the disgusting stimuli and the neutral images has been observed in the acquisition phase. A sentence on this statement has been added in the discussion section of the document

P.18 Line [15 ] In view of the lack of normative data regarding the disgust value and its arousal we can also hypothesize that the difference observed in the valence attracted to the conditioned CS could be partially attributable to the intensity of the disgusting stimuli used in the conditioning procedure.

*3) Armstrong et al. (2017) used 8 different disgusting images depicting feces (3), vomit (3), and rotting food (2) that were selected from the International Affective Picture System (IAPS; Lang, Bradley, & Cuthbert, 2008) and online public sources. The authors used eight different disgusting images (824x618p.) depicting spoiled food (2), waste / body secretions (3), unhygienic environments (3) that have been selected from the DIRTI database (Haberkamp et al., 2017) as US. I guess the eight images in the current study were not disgusting enough for the conditioning. The authors should discuss the disgusting images in the current study compared with the images in the reports by Armstrong et al. (2017) and add the limitation of the study.*

**Response:** We thank reviewer #1 for this comment. Following an exchange with Dr Armstrong about the images and the valence estimated by the participants, it turns out that the images used could for at least two of them be indeed more disgusting than those we used (without having standards since also taken from online resources). The question arose at the time of selection and we had indeed made the choice of moderately disgusting images to prevent visual avoidance. (element on which Dr. Armstrong is working today!). A statement on this reflexion was added in the discussion section of the document.

P.18 Line [15 ] In view of the lack of normative data regarding the disgust value and its arousal we can also hypothesize that the difference observed in the valence attracted to the conditioned CS could be partially attributable to the intensity of the disgusting stimuli used in the conditioning procedure.

*Minor points
4) Page 2, Line 47; The authors should describe what abbreviations of HDCC and LDCC stand for.
5) Page 5, Line 125; The unconditional stimulus and conditional stimulus should be changed into unconditioned stimulus and conditioned stimulus.
6) Page 6, Line 129; The authors should differ classical conditioning from operant conditioning.*

*7) Page 10, Line 233; A grammatical error such as “Regression analyses was” should be corrected at revision.*

**Response:** We thank reviewer #1 for those points. All of the remarks have been taken into account and the manuscript has been consequently modified. Note that reviewer #2 having suggested the removal of “dirt” and retaining only the principle of contamination, the acronyms have been modified throughout the document.

P. 2 Line [7 ] “The present research investigates a counter-conditioning intervention in treating disgust-related emotional responses in two groups of individuals with high (High contamination concerns, HCC, n= 24) and low (Low contamination concerns LCC, n = 23) contamination concerns..”

P.5 Line [ 19] “EC involves the transfer of valence from an unconditioned stimulus to a conditioned stimulus so that the conditioned stimulus acquires the aversive characteristics of the unconditioned stimulus regardless of its relation to the unconditioned stimulus. “

P. 5 Line [ 22] “Even if there is considerable evidence to support, similarly to fear, the role of traditional classical predictive conditioning mechanisms in the acquisition of disgust. »

P.9 Line [25 ] “Then two regression analyses were performed to measure the predictive value of Disgust propensity and sensitivity, contamination concerns (OCI-R contamination, DOCS Contamination, and PADUA Contamination), negative affects (PANAS negative) and anxiety trait scores (STAI B) on US expectancy ratings of the conditioned CS and the negative valence ratings of the acquisition phase..”

*Reviewer #2: It needs to be proof-read carefully. It is intelligible, but with many typos, some of which involve misnaming constructs.*

**Response:** We agree with this comment, the manuscript has been fully proofread by a native English speaker

**Introduction:**

1) *“If” at beginning of abstract is confusing.*
2) *This line is the confusion is a little awkward: “Desensitization to disgust handled by counter-conditioning…” Could the authors just use the term “counter-conditioning.”*

**Response:** We thank reviewer #2 for those comments. Manuscript has been modified accordingly:

P.2 Line [4 ] “Obsessive-compulsive disorder (OCD) has long been considered as an anxiety disorder, disgust is the dominant emotion in contamination-based OCD. »

P.2 Line [ 18] “***Conclusion***: counter-conditioning procedure reduces both expectations and conditioned disgust. »

3) *I would avoid the term “Disgust desensitization.” For many, the term sensitivity refers to how much one is bothered by an emotional state, not how prone they are to experiencing it (the authors note this distinction).*

**Response:** We thank reviewer #2 for this comment we fully agree with. the term desensitization has been replaced throughout the document by the term reduction.

4) *Line 88: Typo?: “there is a compelling argument recent research has pointed out”*

**Response:** We thank reviewer #2 for this comment. We modified the text as follows:

P.4 Line [18] “In addition to these variables, recent research has pointed out the key role of various negative emotions such as shame, embarrassment, frustration, anger and contempt and more particularly disgust in the maintenance of contamination-related OCD [7]. »

5) *Line 97: “previously conceptualized disorders within the exclusive framework of anxiety” should be “disorders previously conceptualized within…”*

**Response:** We thank reviewer #2 for this comment. Manuscript has been modified accordingly

P.4 Line [25 ] “Since the 90s however, authors have examined this emotion further and it is clear today that it is involved in a wide range of disorders previously conceptualized within the exclusive framework of anxiety, such as specific phobias and especially fear of spiders [11], blood phobias and injections [12], post-traumatic stress disorder [13] and more particularly contamination related OCD (C-OCD) [14-15]. »

6) *Paragraph on line 107 too short*

**Response:** We thank reviewer #2 for this comment. This section has been entirely remodeled following the suggestion of your next comment

7) *The paragraph beginning on line 111 needs revision. I found many of these proposed explanations for disgust resisting extinction to be hard to follow and not compelling. I would get right to the findings with taste aversion learning and the work of Garcia. Then transition to evaluative conditioning, which to my knowledge was first studied in taste preference (e.g., the “hedonic shift” of the flavor of sucrose after being paired with poison that causes nausea; see Bouton 2007 for summary). It might help to highlight the relation between distaste and disgust earlier in the introduction (disgust is an emotion that seems to be an elaboration of the distaste oral rejection reflex—same outputs mapped onto more abstract inputs). If the authors wish to argue that disgust is resistant to extinction because of EC, they need to do a better job explaining how EC is involved in disgust. Perhaps bring in Rozin et al. 1986, discuss the transfer of essences as a hedonic shift?*

**Response:** We agree with this comment, the reference to the factors which, in addition to being likely to favor the acquisition of a disgut reaction has been deleted from the introduction, a more targeted paragraph including the notion of "hedonic shift" has been added. We have modified the document as follows:

P.5 Line [12 ] “If it is now well established that anxiety is acquired through conventional Pavlovian conditioning mechanisms., other emotions do not exclusively respond to this type of learning. Despite the small number of studies that have studied disgust in the conceptual framework of conditioning theories (Mason & Richardson, 2010; Armstrong, McLenehan, Kittle & Olatunji, 2014; Olatunji, Forsyth, & Cherian, 2007), qualitative differences with other forms of Pavlovian conditioning have been identified, including the reference to an "hedonic shift" from the unconditional stimulus (US) to the conditional stimulus (CS) (Garcia, Hankins, & Rusiniak, 1974) which might be central. A learning model has been proposed that abnormal disgust reactions can be maintained through the process of evaluative conditioning (EC) [8]. EC involves the transfer of valence from an unconditioned stimulus to a conditioned stimulus so that the conditioned stimulus acquires the aversive characteristics of the unconditioned stimulus regardless of its relation to the unconditioned stimulus.”

8) *The paragraph on line 128 needs to be clarified. The section on EC right now is a little redundant in places. Also, some of the claims about EC and the CR becoming independent of the US-CS relation have been challenged. The description of EC in this paper seems a little outdated.*

**Response:** We thank reviewer #2 for this comment. Paragraph has been completed as follows:

P.5 Line [22] “Even if there is considerable evidence to support, similarly to fear, the role of traditional classical predictive conditioning mechanisms in the acquisition of disgust, recent research has suggested that evaluative conditioning effect (EC) could be central in the learning of disgust [8-21]. Although EC procedure look like Pavlovian conditioning, it differs in being less susceptible to extinction [26-27-28-22]. Whereas prediction is central in Pavlovian conditioning, it’s not the case in EC which is characterized by transfer of affective valence. It has in fact been argued that reactions of disgust are essentially acquired by evaluative conditioning effects: an associative learning process by which a neutral stimulus takes on an affective label following its association with an unconditional stimulus. positively or negatively valued (De Houwer, Thomas, & Baeyens, 2001; Hermans, Vansteenwegen, Crombez, Baeyens, & Eelen, 2002; Hofman, DeHouwer, Perugini, Baeyens, & Crombez, 2010). Evaluative conditioning, by allowing individuals to evaluate both new and familiar emotional stimuli (eg, whether something is liked or not, disgusting or desirable) thus facilitates the role of disgust not only as a survival mechanism (Olatunji , Forsyth, & Cherian, 2007) but also as a fundamental adaptation mechanism of humans to their environment.”

**Methods:**

9) *I would suggest removing “dirt” from the group names—just the term contamination should suffice.*

**Response:** Following the reviewer’s suggestion, the term « dirt » has been removed throughout the document and the acronym changed from HDCC and LDCC to HCC and LCC.

***Power***

10) *DPSS is propensity and sensitivity scale, not propulsion and sensibility.*

**Response**: We thank reviewer #2 for this comment. Text has been modified, typo have been checked.

P.18 Line [21 ] “Yet it has been previously shown that disgust propensity was highly correlated with OCD »

11) *The ratings do not seem retrospective, because they don’t ask about feelings of disgust in the past, during the task. This is fine; I would just suggest not calling them “retrospective” ratings.*

**Response:** We thank reviewer #2 for this comment. We modified the text as follows:

P.9 Line [ 6] “At the end of each phase, participants use the empirical valence scale (EVS) to score the disgust felt for CS “How much do you find this picture disgusting?” and how much they expect the disgusting US to follow CS “How much do you expect this picture to be followed by a disgusting picture”.

**Results:**

12) *Error in table 1: Genre % Female*

**Response:** We are sorry but didn’t get what reviewer #2 means here.

13) *The term valence is confusing in this section. The measure is called the empirical valence scale, but it is used here to measure disgust, rather than the bipolar valence dimension of affect, so the outcome should be referred to as disgust not valence.*

**Response:** We thank reviewer #2 for this comment we fully agree with. We modified the text replacing “valence” by “disgust” whenever necessary.

14) *The figures could use a y-axis label. It is clear that it is the ratings, but with the different min/max it is a little confusing. Also, I might try to produce better figures, as these figures have no error bars, have periods after the Y-axis values, and have counter-conditioning misspelled.*

**Response:** We thank the Reviewer 2 for these suggestions. Accordingly, we adapted the figure 1 in the revised version of the manuscript, dividing the figure in expectation and disgust as have suggested reviewer #3.

HCC Group

| Phase | CS+ | CS- |
| --- | --- | --- |
| HABITUATION | 31.1364 | 41.8182 |
| ACQUISITION | 91.1364 | 2.09091 |
| COUNTER CONDITIONING | 31.5909 | 7.31818 |

HCC Group

| Phase | CS+ | CS- |
| --- | --- | --- |
| HABITUATION | 31.1364 | 41.8182 |
| ACQUISITION | 91.1364 | 2.09091 |
| COUNTER CONDITIONING | 31.5909 | 7.31818 |

LCC Group

US Expectancy

| Phase | CS+ | CS- |
| --- | --- | --- |
| HABITUATION | 35.6957 | 39.7826 |
| ACQUISITION | 94.6957 | 1.26087 |
| COUNTER CONDITIONING | 11.3913 | 10.3478 |

ratings of the CS+ and CS- by Groups

LCC Group

| Phase | CS+ | CS- |
| --- | --- | --- |
| HABITUATION | 1.26087 | 1.52174 |
| ACQUISITION | 2.78261 | 1.26087 |
| COUNTER CONDITIONING | 1 | 1 |

HCC Group

Valence

| Phase | CS+ | CS- |
| --- | --- | --- |
| HABITUATION | 5.18182 | 5.95455 |
| ACQUISITION | 11.1818 | 1.81818 |
| COUNTER CONDITIONING | 8.22727 | 1.81818 |

Figure 1. Direct evaluation and US expectation

*Note*: *HCC: High contamination concerns group, LCC : Low contamination concerns group.*

15) *Line 286: I did not follow this: “A t-test 287  reveals that only conditioned CS (CS +) was significantly assessed as causing disgust in the HDCC 288  group who associated the aversive properties of the CS from the acquisition phase t(43)=5.17, p=.02”
     Perhaps use holm-bonferroni procedure to increase power?*

**Response:** We agree that this sentence is confusing. We revised it as follows:

P. 14 Line [15 ] « A t-test reveals that the HCC group reported experiencing more disgust to the conditioned CS (CS +) following the acquisition stage t(43)=5.17, p=.02, 95% CI [-.47; 1.5] (see Table 2) »

16) *In the methods section, I did not complete understand this sentence: “Regression analyses was then conducted through a step by step procedure including the whole of the 7 sub-dimensional factors assessed by scales. “*
17) *Then, in the results section, I did not follow the related analyses. It would help to be more specific about the dependent variable in these analyses.*

**Response:** we thank the reviewer 2 for this comment. Accordingly, we modified these two sentences in both the statistical plan section and the results section of the revised manuscript:

*Statistical plan*

P. 9 Line [25 ] *“Then two regression analyses were performed to measure the predictive value of Disgust propensity and sensitivity, contamination concerns (OCI-R contamination, DOCS Contamination, and PADUA Contamination), negative affects (PANAS negative) and anxiety trait scores (STAI B) on US expectancy ratings of the conditioned CS and the negative valence ratings of the acquisition phase. Furthermore, a Bonferroni adjusted significance level of p < 0.003 was applied in the regression analyses to control for problems in multiple testing.”*

*Results*

P. 9 Line [25 *“Two regressions analyses were performed entering simultaneously as predictors the Disgust propensity and sensitivity scores, as well as contamination concerns (OCI-R contamination, DOCS Contamination, and PADUA Contamination), negative affects (PANAS negative) and anxiety trait scores (STAI B) on the two following dependent variables: US expectancy ratings of the conditioned CS and the negative valence ratings of the acquisition phase. Together, the seven predictor variables explained a significant proportion of the variance in expectancy acquisition of the conditioned CS (r^2^ = .47, F(1,40) = 11.5, p =.002) and the negative valence acquisition (r^2^ = .45, F(1,40) = 10.62, p=.002) assessed with the EVS. The unique significant predictor for evaluative acquisition score was Disgust sensitivity (β = 1.52, t = 3.4, p = .002). Whereas, expectation ratings after counter conditioning procedure are only predicted by anxiety scores as assessed by the STAI B (r^2^ = .78, F(5,36) = 11.21, p <.001).*

**Discussion:**

18) *The authors claims that “From a clinical point of view, exposure-based treatment incorporating counter-conditioning would thus be more effective than simple exposure treatment by addressing not only the predictive value  of the unconditioned stimulus occurrence but also by modifying the valence attributed to the conditioned stimulus.” However, as they note in the limitations, there is not direct comparison to an extinction procedure. In the limitations, I would be more explicit about needing to replicate the effect with an extinction comparison group, as other researchers have done.*

**Response:** We totally agree with this statement, we have added the following sentence at the end of the discussion section:

P. 20 Line [5 ] “Nevertheless, it will be essential to effectively observe the benefit of counter conditioning by comparing it directly with a simple exposure procedure in order to be able to draw firm conclusions.”

19) *One other limitation is statistical power. The group sizes are relatively small for detecting between-groups differences. This limitation applies to much of the conditioning literature.*

*The authors note the need for other dependent variables in their conditioning research. They might consider eye tracking: e.g., Armstrong, T., Engel, M., Press, T., Sonstroem, A., & Reed, J. (2019). Fast-forwarding disgust conditioning: US pre-exposure facilitates the acquisition of oculomotor avoidance. Motivation and Emotion, 43(4), 681-695.*

**Response:** We are totally agreed with this comment. We added those points in the limitations section of the revised manuscript:

P. 20 Line [16 ] “Furthermore, it is important to note that the sample size of the included participants in the study was relatively small although sufficient to demonstrate significant effects of a counter conditioning procedure on disgust extinction in OCD patients with high contamination concerns. Therefore, our findings need to be replicated in larger samples.”

P.21 Line [5 ] “In addition, self -report as common variance method may have inflated the correlations among the study variables. Future studies would benefit from using less direct and less subjective observation techniques such as emotional priming, behavioral avoidance task, eye tracking or physiological measures.”

Reviewer #3:

**Keywords**

*1) C-OCD is not a clear acronym.*

**Response:** We agree with this comment. We have modified the keyword accordingly.

P.2 Line [21] “Keywords: Disgust, Contamination-based OCD, Contamination fear, Evaluative conditioning, counter conditioning”

**Introduction**

*2) Line 85 - I believe that there is a typo here with the word “if” - the sentence is meaningless with it.*

**Response:** The sentence was modified accordingly

P.4 Line [14 ] “It has been proposed that the main reasons of ERP’s failure include patients’ motivation disability in reducing rituals and the presence of comorbid disorders, like depression or avoidant personality, specifically in contamination-based OCD [6]. “

*3) Throughout the manuscript, authors use the word “concerns” to name the phenomena that they are observing/describing. My question here is: why not calling it symptoms? Even individuals that do not fulfill diagnostic criteria for OCD could have symptoms.*

**Response:** We thank reviewer 3 for this comment. Following many relevant papers cited throughout the manuscript, we decided to use the term « concern » rather than « symptoms » to distinguish analogue samples from individuals with diagnosed OCD ant to avoid over-pathologizing of (potential) vulnerable samples. However, we totally share the view that OC symptoms are dimensional rather than categorical in frequency and severity (see for example Abramowitz et al. (2009) cited in our paper).

*4) Line 130 - authors should better characterize evaluative conditioning in relation to classic conditioning. What are the differences? What are the specific characteristics of EC?*

**Response:** We thank reviewer 3 for this comment. Paragraph has been completed as follows:

P.5 Line [22 ] “Even if there is considerable evidence to support, similarly to fear, the role of traditional classical predictive conditioning mechanisms in the acquisition of disgust. Recent research has suggested that evaluative conditioning effect (EC) could be central in the learning of disgust [8-21]. Although EC procedure look like Pavlovian conditioning, it differs in being less susceptible to extinction [26-27-28-22]. Whereas prediction is central in Pavlovian conditioning, it’s not the case in EC which is characterized by transfer of affective valence. It has in fact been argued that reactions of disgust are essentially acquired by evaluative conditioning effects: an associative learning process by which a neutral stimulus takes on an affective label following its association with an unconditional stimulus. positively or negatively valued (De Houwer, Thomas, & Baeyens, 2001; Hermans, Vansteenwegen, Crombez, Baeyens, & Eelen, 2002; Hofman, De Houwer, Perugini, Baeyens, & Crombez, 2010). Evaluative conditioning, by allowing individuals to evaluate both new and familiar emotional stimuli (eg, whether something is liked or not, disgusting or desirable) thus facilitates the role of disgust not only as a survival mechanism (Olatunji , Forsyth, & Cherian, 2007) but also as a fundamental adaptation mechanism of humans to their environment.”

*5) Line 136 - the sentence: “Targeted disgust-based exposure has resulted in improvement over traditional exposure based on anxiety in the treatment of blood-injection injury phobia for both symptom reduction and global health measures“ needs to be referenced.*

**Response:** Indeed! The reference was added in the manuscript.

P.6 Line [7 ] “Targeted disgust-based exposure has resulted in improvement over traditional exposure based on anxiety in the treatment of blood-injection injury phobia for symptom reduction [31]. »

Olatunji BO, Ciesielski BG, Wolitzky-Taylor KB, Wentworth BJ, Viar MA. Effects of experienced disgust on habituation during repeated exposure to threat-relevant stimuli in blood-injection-injury phobia. Behav Ther. 2012 Mar;43(1):132-41.

*6) Line 145 “only few studies”. Cite those studies.*

**Response:** We thank reviewer 3 for this comment. The paragraph has been modified as no study to our knowledge has examined if a counter conditioning procedure might reducee learned disgust reactions.

P.6 Line [14 ] « In this study we decided to focus on the potential utility of a counter conditioning procedure in reducing learned disgust reactions. The counter-conditioning procedure implies pairing a conditioned stimulus (CS) with an unconditional stimulus (US) of an opposite valence from the original US. This technique could be effective for two reasons. First, by reducing the effects of the negative evaluation attributed to an item by granting it the valence of the new representation. Secondly by reducing the expectation of the conditioned response as it can be through simple exposure techniques by replacing the old aversive association. Several studies using self-reported measures, emotional priming tasks, as well as behavioral tasks have shown positive effects of counterconditioning on disgust-related evaluative learning [32-33]. However, despite the fact that the participants underwent a conditioning procedure with disgusting unconditional stimuli, these authors did not directly assess the disgusting aspect of their conditioned stimuli, limiting the scope of the conclusions that can be drawn from it. »

».

*7) Counter-conditioning has a hyphen - authors should standardize this term throughout the entire manuscript. The title, for example, has no hyphen.*

**Response:** We totally agree with this comment, the term has been standardized.

*8) Did the authors know the work by Koizumi and colleagues (2016) in Nature Human Behavior showing counter-conditioning in fear with fMRI? It is a possible reference that could be used in the introduction, or in the discussion section, for future studies with counter-conditioning in disgust.
Koizumi A, Amano K, Cortese A, Shibata K, Yoshida W, Seymour B, Kawato M, Lau H. Fear reduction without fear through reinforcement of neural activity that bypasses conscious exposure. Nat Hum Behav. 2016;1:0006. doi: 10.1038/s41562-016-0006. Epub 2016 Nov 21. PMID: 28989977; PMCID: PMC5628620.*

**Response:** We thank reviewer 3 for this important precision. This article is indeed very interesting for this subject and we were not aware of it. Especially as various analyzes have highlighted the importance of the consciousness of contingency as a prerequisite for the establishment of an evaluative conditioning. The reference and the subject were added in the discussion section of the document.

P.21 Line [5 ] “In addition, self -report as common variance method may have inflated the correlations among the study variables. Future studies would benefit from using less direct and less subjective observationtechniques such as emotional priming, behavioral avoidance task, eye tracking or physiological measures. Moreover, a recent study using *Fmri* in fear counter-conditioning, has pointed out that implicit association could reduce the emotion generated by a conditioned stimulus [57], and could prove an interesting perspective of research.

**Materials and Methods**

*9) Lin 166 - The sample needs to be better characterized: who were these 162 “lay people’? How they were contacted? They were accessed via the internet or in person? There is a bias in the selection, for example, individuals from the university or from the hospital?
How did you get the final sample of 25 and 23 participants in each group? It was arbitrarily?*

**Response:**

In response to the reviewer’s comment we added

P.21 Line [5 ] “Following Amstrong et al.’s procedure (2017), one hundred and sixty-two undergraduate students from Paul Valery Montpellier 3 University were tested using Padua's inventory contamination subscale (PI-C) [37] to identify people with low and high contamination-related concerns. In line with other studies [38-39-40], individuals with scores greater than 13 (77: 47.53% of the sample tested) were considered eligible for the group with high concerns about dirt and contamination (HCC) and individuals with scores below 7 (42: 27.92% of the sample tested) were considered eligible for the group with low concerns regarding dirt and contamination (LCC). In order to maximize the distinction between groups we then started recruiting participants with highest and lowest scores. Given the timeline of our study, our final sample comprised 25 participants in the HCC group and 23 participants in the LCC group. »

*10) Line 195 - there is a ref with the author/date system *Haberkamp et al., 2017).*

**Response:** We thank reviewer 3 for this comment. We have modified the text accordingly.

*11) Line 204 - 2016 should be within parenthesis.*

**Response:** We thank reviewer 3 for this comment. We have added parenthesis accordingly.

*12) Line 206 - ITI isn’t the acronym for inter trail interval?*

**Response:** We thank reviewer 3 for this comment. An error in the manuscript that was corrected.

*13) Why, in the habituation phase, there is a long interval (from 12 to 18 seconds)? In fMRI studies, they are used for the BOLD signal returns to its baseline. Is there a reason for this behavioral study?*

**Response:** We thank reviewer 3 for this comment. The experiment was adapted from Armstrong and Olatunji’s work, so we modeled our procedure on theirs. But indeed, a lower ITI should have been sufficient to allow not to associate CS- with potentially previous disgusting image.

**Statistical plan**

*14) first the authors mention that analyses were conducted separately, so they didn’t correct for multiples comparisons (that’s ok), but after that, they mention that ratings evolution between acquisition and CC needed a within-subject factor. This was not clear to me. The ANOVA model that included stage is not a repeated measures ANOVA?*

**Response:** The ANOVA model that included stage is a repeated measures ANOVA with Group (HDCC; LDCC) as between-subject factor, and CS (CS+; CS-) and Stage (acquisition, extinction) as within-subject factors. To avoid any confusion, we have specified this point as follows in the revised version of the manuscript:

P.9 Line [13]“ Consequently, we did not correct for multiple comparison across phases. For the disgust ratings and the US expectancy ratings, we used mixed-model repeated analysis of variance with Group (HCC; LCC) as between-subject factor and CS (CS+; CS-) as the within-subject factor. Significant CS x group interaction were followed by independent sample t-test in order to compare between groups the ratings of the CS+ and the CS- separately. To observe the evolution of the ratings between acquisition and counter conditioning stages, a same statistical design was applied adding the within-subject factor of phase (acquisition, counter conditioning) to the previous ANOVA model that included the CS and group factors.”

*15) Which statistical software or package was used to run the analysis?*

**Response:** The analysis was conducted with SPSS. A sentence was added in the statistical plan to precise it.

P.9 [13 « All statistical analyses were performed with IBM SPSS software version 24.0. Following the work lead by Armstrong and Olatunji in 2016, analyses were conducted separately for each phase of the procedure (habituation, acquisition, counter conditioning (CC)).”

*16) Also, in lines, 213, 208, and 205 authors use the word ‘phase’ to name the section of the experiment, but in the statistical plan, they changed it to ‘stage’. This confounds the reader. Only one nomenclature should be used throughout the entire manuscript.*

*17) Line 231 - typo: two final dots.*

*18) Line 241 - all p-values are written with three decimals, but the last one in the sentence is with only two (this can need to be corrected in the tables as well).*

**Response:** We thank reviewer #3 for those observations. We have standardized the terms used accordingly, using “phase” to name the experiment section, all typo have been corrected and text has been unified in results presentation.

**Results**

*19) Line 241 - there is an extra space before the closing parenthesis.*

*20)I would like to see boxplots for each variable in Table 1 and 2 to see their distributions. Do they have normal (or approximately normal) distribution?*

**Response:** No normal distribution was considered when absolute values for skewness and kurtosis were greater than 3 and 10, respectively (Weston & Gore Jr., 2006).

As you can see below, clinical variables in Table 1 for each group were normally distributed. However, data for valence ratings and US expectancy evaluation were normally distributed or approximately normally distributed for certain. Here are the values for skewness and kurtosis, as well as the boxplots for each variable in Table 1 and 2:

Clinical characteristics (Table 1) for the HCC Group:

Clinical characteristics (Table 1) for the LCC Group:

Valence ratings and US expectation per phases (Table 2) for the HDCC Group:

*Note: Hab= habituation; Acq=Acquisition; CC= counter conditioning; CS+ = CS1; CS- = CS2*

Disgust ratings:

Us expectation:

Disgust ratings and US expectation per phases (Table 2) for the LDCC Group:

Us expectation:

Disgust ratings*:*

*21) Table 1 - why the HDCC group has n = 22 participants in the table? I don’t know if I missed something but in the abstract (and at the beginning of the methods) authors mention that N = 25. How (and why) those three participants were excluded? Or this is a typo?*

**Response:** We thank reviewer #3 for this observation A typo corrected.

*22) Table 2 - there is a missing SD in the valence (CS-) of LDCC group. Also, since one of the main goals of this research is the between-group comparison, I believe that Table 2 should be re-oriented (eventually broken into two tables): groups must be at the columns, side-by-side (eventually with p-values in the third column as well), with expectation in one block and valence in the other (if authors prefer, they could slipt two tables, one for expectation and the other to valence). The experiment phases should be reoriented to the lines.*

**Response:** We thank the reviewer 3 for this suggestion. Accordingly, we revised the table 2 as follows:

|  |  | **HCC (N=24)** | **LCC (N=23)** |
| --- | --- | --- | --- |
| **Expectation** |  |  |  |
| Habituation | CS+ | 31.13 (32.10) | 35.69 (30.79) |
|  | CS- | 41.81 (33.98) | 39.78 (28.61) |
| Acquisition | CS+ | 91.13 (11.01) | 94.69 (14.37) |
|  | CS- | 2.09 (2.36) | 1.26 (1.25) |
| Counter conditioning | CS+ | 31.59 (32.42) | 11.39 (20.86) |
|  | CS- | 7.31 (15.56) | 10.34 (20.85) |
| **Valence** |  |  |  |
| Habituation | CS+ | 5.18 (14.75) | 1.26 (1.25) |
|  | CS- | 5.95 (14.75) | 1.52 (1.72) |
| Acquisition | CS+ | 11.18 (18.09) | 2.78 (5.06) |
|  | CS- | 1.81 (2.10) | 1.26 (1.25) |
| Counter conditioning | CS+ | 8.22 (16.46) | 1 (0) |
|  | CS- | 1.81 (2.10) | 1 (0) |

*23) Figure 1 - the acquisition phase is not named. Also, authors must include error bars for the reader to visualize the variability. It could be CI, SD, or SE.*

*24) Why not also inserting a figure for CS-? Visualizing it and comparing it to Figure 1 would be much easier than interpreting the tables. If the authors could insert the CS- in Figure 1 would be even better (since there is not too much information, of course). I also suggest breaking Figure 1 into two figures - one just for Valence, the other only for expectancy,*

**Response:** We thank the Reviewer 3 for these suggestions. Accordingly, we adapted the figure 1 in the revised version of the manuscript as follows:

*25) Overall, the results are very long and tiring for the reader - I would recommend the authors to give more emphasis on the tables and, mainly on Figures. Explaining the results thought the figures would be much better.*

**Response:** We agree with this comment. We have effectively referred the reader to tables and figures whenever possible.

*26) Line 309 - check parenthesis and comma. Also, LDCC is writing as LCC (lots of typos throughout the manuscript!). This makes it even harder for the reader to understand the long results and the many analyses performed.*

**Response:** We agree with this comment, entire manuscript have been carefully checked for this kind of typos. *27) Lines 306 and 309 - difference averages don’t need to be written in the text (as in this case).*

**Response:** We deleted this information in the revised version of the manuscript.

*28) Lines 335-341 I couldn’t understand if there were significant associations. In the Methods section there is no reference to correlation. If authors performed correlations, a table showing each correlation coefficient (and p-value) could be inserted as supplementary files.*

**Response:** We agree with this comment. This paragraph has been removed from the manuscript.

**Discussion**

*29) Line 370 - It is a new paragraph? I don’t see why.*

**Response:** We thank reviewer 3 for this comment and have linked this paragraph with the previous one.

*30) The difference between using faces vs abstract pictures should be further explored in the discussion - what are the authors’ hypotheses about it? Why changing stimuli could lead to differences?*

**Response:** We thank reviewer 3 for this comment. In a previous pilot study conducted to evaluate the impact of different neutral and unconditional stimuli on disgust evaluative conditioning, we have observed that the association was stronger with abstract pictures than with faces.

*31) Line 396 - the authors could explain what they meant with the sentence “This knowledge must rely on associative network.”*

**Response**: We thank reviewer 3 for this comment. Evaluative conditioning, by allowing individuals to evaluate both new and familiar emotional stimuli (eg, whether something is liked or not, repulsive or desirable) the emotional status of a stimulus is already (consciously or not) known by the individual. However, we agree with the confusion created by this sentence and so removed it from the reviewed manuscript.

*32) Lines 408-415 - is it possible that more trails (or even a second-round) of CC could be effective to turn the negative valence off?*

**Response**: We thank reviewer 3 for this comment. Indeed, some works have observed that more exposure trials were necessary to reduce disgust than fear, that disgust would have a lasting form of learning, it would be modifiable but difficultly (Olatunji, Forsyth, Cherian, 2007). We could thus suggest that more trials could be effective to turn the valence off. Experimental tasks have to be done. A sentence has been added in the perspective section of the document.

P. 19 Line [16 ] “Moreover, some works have observed that more exposure trials were necessary to reduce disgust than to reduce fear, that disgust would have a longer lasting form of learning, it would be modifiable but with difficulty [24]. We could thus suggest that more counter-conditioning trials could be effective to turn the valence off.»

*33) Lines 416-419 - This last paragraph could also be extended: authors could give an example of what would it be to incorporate counter-conditioning in the clinical practice (illustrating in word what is to address the predictive value and to modify the valence).*

**Response**: We thank reviewer 3 for this suggestion. Some example were added at the end of the discussion section.

P.20 Line [1 ] “From a clinical point of view, exposure-based treatment incorporating counter conditioning would thus be more effective than simple exposure treatment by addressing not only the predictive value of the unconditioned stimulus occurrence but also by modifying the disgust attributed to the conditioned stimulus (for example, wearing a beloved one’s clothing during exposure, smelling or tasting something one likes, hearing a loved music…). Nevertheless, it will be essential to effectively observe the benefit of counter conditioning by comparing it directly with a simple exposure procedure in order to be able to draw conclusive results.»
