# Supplementary material for: Acquisition and maintenance of disgust reactions in an OCD analogue sample: Efficiency of extinction strategies through a counter-conditioning procedure
Source: PLoS One. 2021 Jul 14;16(7):e0254592. doi: 10.1371/journal.pone.0254592 (PMC8279387; doi:10.1371/journal.pone.0254592)
Supplement: Attachment — Submitted filename: response to reviewer .docx [file pone.0254592.s004.docx]

03 of June, 2021

Ref PONE-D-20-33124

Dear Editor, Dear reviewers

We have carefully considered the Reviewers’ comments and have revised the manuscript accordingly. Below we provide a detailed explanation of how the manuscript was modified.

Reviewer #3:

Authors have to choose only one way of reporting counter conditioning: with or without the hyphen, all together as in “counterconditioning”, and apply it through the entire manuscript.

**Response:** We thank reviewer#3 for this comments. Manuscript has been modified accordingly.


Do not start the introduction with an acronym.

“Cognitive Behavioral Therapy” does not need capital letters.
Pavlovian Conditioning – one time is written with capital letters and the second time only Pavlovian is with a capital letter (the second is right).

Pg 5, line 13 – there is an extra dot.
Pg 5, line 15 – refs should be [21-24] not [21- 23-24]
Pg 5, line 24 – ref should be [8, 21] not [8-21]
In the text, PI should be PI-C (following the acronym that first appear in the text).
Pg 7, line 21 there is an extra parenthesis, or parenthesis is lacking. In the same sentence – there is no need to start the scale’s name with capital letters.
Too many typos for a revised version of the manuscript! The authors should review the entire text.

**Response:** We thank reviewer#3 for those comments. Manuscript has been modified accordingly.

*Methods*

Nice that authors incorporated the Bonferroni correction – however that rationale for using it was not described – i.e. how many comparisons are the authors controlling for?
The Bonferroni was used only for the regressions or the other analyses as well? Please be more specific and clear regarding this issue.

**Response:**

We chose to use a Bonferroni correction only in our two regression analyses to correct for multiple comparisons. Since we ran two regression analyses in which we had 7 predictor variables (i.e. Disgust propensity and sensitivity, contamination concerns (OCI-R contamination, DOCS Contamination, and PADUA Contamination), negative affects (PANAS negative) and anxiety trait scores (STAI B)), we simply divided our α value (.05) by the number of simultaneous multiple comparisons. We have seven predictors in each of our regression analyses, so we divided our α value by 7 to get a more realistic significance level (i.e. .007).

We have added this explanation in the statistic plan section of the revised version of the manuscript:

*“Then two regression analyses were performed to measure the predictive value of Disgust propensity and sensitivity, contamination concerns (OCI-R contamination, DOCS Contamination, and PADUA Contamination), negative affects (PANAS negative) and anxiety trait scores (STAI B) on US expectancy ratings of the conditioned CS and disgust ratings of the acquisition phase. Since we have seven predictors in each of our two regression analyses, we chose to use a Bonferroni correction to correct for multiple comparisons. For this, we divided our α value (i.e. .05) by 7 to get a more realistic significance level (i.e. .007).*

*All analyses were conducted with a significance threshold of α ≤ .05, two-tailed.”*

*Results*

Pg 11, line 8 – an extra parenthesis in the text.

**Response:** We thank reviewer#3 for this comment. Manuscript has been modified accordingly.


Table 2 – the information between parenthesis is SD? Why there are no between-group comparisons in this table? This should be informative for the reader.

**Response:** the information between parenthesis is indeed SD. This information is yet mentioned in the title of the table 2: *“Mean (SD) of direct evaluation on US expectation and valence ratings on Empirical valence scale (100 points)”*

Accordingly, we added the between-group comparisons in the table of the revised version of the manuscript:

***Table 2.*** Mean (SD) and between-group comparisons of direct evaluation on US expectation and valence ratings on Empirical valence scale (100 points)

|  |  | HCC (N=24) | LCC (N=23) | Test Statistic | Sig., effect size |
| --- | --- | --- | --- | --- | --- |
| Expectation |  |  |  |  |  |
| Habituation | CS+ | 31.13 (32.10) | 35.69 (30.79) | F (1,43) = .02 | p = .87, n^2^_p_ = .001 |
|  | CS- | 41.81 (33.98) | 39.78 (28.61) |  |  |
| Acquisition | CS+ | 91.13 (11.01) | 94.69 (14.37) | F (1,43) = .50 | p = .48, n^2^_p_ = .01 |
|  | CS- | 2.09 (2.36) | 1.26 (1.25) |  |  |
| Counter conditioning | CS+ | 31.59 (32.42) | 11.39 (20.86) | F (1,43) = 2.32 | p = .13, n^2^_p_ = .07 |
|  | CS- | 7.31 (15.56) | 10.34 (20.85) |  |  |
| DISGUST |  |  |  |  |  |
| Habituation | CS+ | 5.18 (14.75) | 1.26 (1.25) | F (1,43) = .03 | p = .17, n^2^_p_ = .04 |
|  | CS- | 5.95 (14.75) | 1.52 (1.72) |  |  |
| Acquisition | CS+ | 11.18 (18.09) | 2.78 (5.06) | **F (1,43)= 4.75** | **p = .03, n^2^_p_ = .10** |
|  | CS- | 1.81 (2.10) | 1.26 (1.25) |  |  |
| Counter conditioning | CS+ | 8.22 (16.46) | 1 (0) | **F (1,43)= 12.98** | **p = .001, n^2^_p_ = .23** |
|  | CS- | 1.81 (2.10) | 1 (0) |  |  |

Also, I couldn’t understand the 1 and (0) in the last two lines of the LCC column.
Authors could review this information, Pg 14, line 16: t(43)=5.17, p=.02, 95% CI [-.47; 1.5]. If the CI is crossing zero, then it should not be significant.
What information the IC was meant to show in the results (they only appear in the t-test comparison), please clarify.

**Response:** As mentioned above, data are presented as follows: mean (SD). Hence, 1 is the mean of Disgust ratings for the LCC group, and (0) is SD. These values are also mentioned in the figure 1.

We thank the reviewer 3 for this remark. This was a typo. Accordingly, we review this information as follows:

*« A t-test reveals that the HCC group reported experiencing more disgust to the conditioned CS (CS +) following the acquisition stage t(43)=5.17, p=.02, 95% CI (3.69; 34.63] (see Table 2) »*

Most frequently, confidence intervals (CI) are used to bound the mean or standard deviation, but also for the differences between populations (= CI for group means). CI for group means are ranges of values that are likely to contain the true mean of each population. In this context, the confidence interval is used to assess the estimate of the true population mean for each group. Hence, the CI for group means can be available only for t-tests in our statistical analyses because we mainly used these means comparisons tests to probe the interaction terms coming from the repeated analysis of variance.

The results section is very long and the reader gets tired of reading. It is hard to follow so many analyses – Figure 1 should guide the results. Much more easy to interpret the Figure than the pages and pages of p-values and different comparisons. Authors should think of a way of passing the information that is more direct and clear and presented it in the new version of the manuscript.

**Response:** To facilitate the reading of the results, we have referred the reader as much as possible to Figure 1, as well as to the inter-group comparisons table (table 2) in the revised version of the manuscript.

Also about figure 1 - HCC is on the left in the upper part, but at the right in the bottom part. Standardize this to facilitate the reader’s life!

**Response:** We thank the reviewer 3 for this remark. Accordingly, we standardize this point.

At the end of the paragraph about the regression analyses is was not clear to me why de disgust sensitivity was represented with Beta, t, and p-value, whereas the STAI-B has r², f, and p-value. Both analyses shouldn’t have the same parameters (as they were similar). What is the beta value of STAI-B?

**Response:** We are very confusing for this mistake. Accordingly, we reviewed this section as follows:

*Whereas, expectation ratings after counter conditioning procedure are only predicted by anxiety scores as assessed by the STAI B (β = .89, t = 3.36, p = .002).*

*Discussion*

Pg 17, line 26 – there is an open parenthesis that is not closed. The third time that this happens in the manuscript.

**Response:** We thank reviewer#3 for this comment. Manuscript has been modified accordingly.

P 18, line 10 “This is also consistent with some work suggesting that attentional biases and pre-sensory learning would be an important trait in people with OCD and could foster learning and facilitate the creation of disgust conditioned response, whereas people who do not have OCD may not perceive anything.” Authors could cite this work. Is it ref #33?

**Response:** We thank reviewer#3 for this comment. Indeed, the quote is taken from the work of MC Kay (2006), the manuscript has been modified for clarity as follow:

P.18, l.9 This is also consistent with some work suggesting that attentional biases and pre-sensory learning would be an important trait in people with OCD and could foster learning and facilitate creation of disgust conditioned response, whereas people who do not have OCD may not perceive anything [33]. This may increase their susceptibility to disgust.

Regarding the previous response, I’m still not satisfied with the boxplots shown in the pdf – many of them were cut, the x-axis title was not readable.

**Response:** indeed, the layout has shifted the box plots. We apologize and represent the graphics to you.

Clinical characteristics (Table 1) for the HCC Group:

Clinical characteristics (Table 1) for the LCC Group:

Valence ratings and US expectation per phases (Table 2) for the HDCC Group:

*Note: Hab= habituation; Acq=Acquisition; CC= counter conditioning; CS+ = CS1; CS- = CS2*

Disgust ratings:

Us expectation:

Disgust ratings and US expectation per phases (Table 2) for the LDCC Group:

Us expectation:

Disgust ratings*:*

Most importantly, in the tables presented in their response, there are 24 participants for the LCC group and 24 for the HCC group, whereas in table 1 of the main manuscript the number of the LCC is 23 and the main text in the “participants section” the authors wrote that their HCC sample was 25. This is confusing!

**Response:** We thank reviewer #3 for his alertness. indeed, the error was not in the tables or the introduction but in the participant’s section. The error has been corrected.
